# Platelets—Disarmed guardians in the fight against the plague

**DOI:** 10.1111/jth.15063

**Published:** 2020-12-03

**Authors:** Alice Assinger

**Affiliations:** ^1^ Department of Vascular Biology and Thrombosis Research Center of Physiology and Pharmacology Medical University Vienna Austria

Platelets represent the first line of defense upon injury, acting as large army of anucleated cells that wall up damaged vessels to prevent blood loss and to protect the host against invading pathogens. At the same time platelets, via release of soluble factors, sound the alarm to recruit leukocytes and put the immune‐defense troops on standby.^1^

The multiplicity of platelets, their sensitive nature, and quick reply to changes in the environment turn them into a very dangerous opponent for any invader. Therefore, enemies must find a way to outflank platelets to successfully complete their sinister missions.

More than 6000 years ago, one such powerful invader, *Yersinia pestis*, emerged from enteric bacterial ancestors. Known as the Black Death, the plague caused by *Y pestis* spread quickly for centuries, killing millions. It is one of the deadliest diseases, and plague outbreaks represent the most notorious epidemics in history.^2^

Transmitted by fleas from rodent reservoirs, *Y pestis* represents a highly sophisticated pathogenic bacterium with an impressive repertoire of combat gear. It possesses a complex set of virulence determinants, including classical pathogen‐associated molecular patterns, the *Yersinia* outer‐membrane proteins (Yops), the broad‐range protease Pla, and iron capture systems. They all play critical roles in the molecular strategies of *Y pestis* warfare to subvert the human immune system, allowing unrestricted bacterial dissemination and replication.^2^

Moreover, like other infamous invaders, including *Chlamydia*, *Pseudomonas*, *Salmonella*, *Shigella*, and *Vibrio*, *Y pestis* is equipped with a special weapon, the type III secretion system (T3SS), which is essential for its pathogenicity. The T3SS system represents an ingenious protein nano‐syringe to hijack eukaryotic cells via injection of virulence effectors directly into host cytosol. Contact of the needle with a host cell triggers the T3SS to start secreting.^3^ However, the trigger and the exact way in which effectors enter the host remain largely a mystery.

At the end of the day, however, all this fancy equipment becomes useless when the first line of defense can withhold the attack. Therefore, not only the immune system but also the hemostatic system need to be outwitted to favor intracellular spread of the bacterium.

In this issue, Palace et al elegantly demonstrate how *Y pestis* found a way to escape entrapment in thrombi (schematic overview in Figure 1).^4^

**FIGURE 1:**
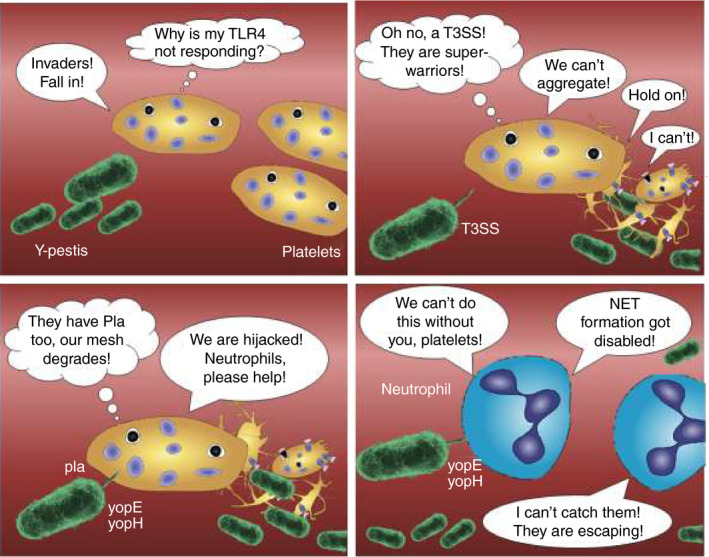
*Yersinia pestis* outwits platelets by (1) evading recognition via Toll like receptor 4 (TLR4), (2) inhibiting platelet aggregation via type III secretion system (T3SS) and protease Pla, and (3) impairing cytoskeletal rearrangements in platelets via YopE and YopH. This leads to destabilization of thrombi and diminished platelet‐neutrophil cross talk. Escape of bacteria is further fostered by *Yersinia pestis*‐mediated inhibition of neutrophil phagocytosis and neutrophil extracellular trap (NET) formation

They found that the presence of *Y pestis* inhibits platelet aggregation in response to thrombin and renders thrombi unstable, leading to their disaggregation. The inhibiting effects on platelet aggregation were dependent on functional Pla protease, which is also responsible for the fibrinolytic properties of *Y pestis*. This surface‐exposed, transmembrane β‐barrel protease exhibits a complex array of interactions with the hemostatic system: It favors fibrinolysis by direct plasminogen and urokinase activation while inactivating the serpins plasminogen activator inhibitor‐1 and α2‐antiplasmin as well as the thrombin‐activatable fibrinolysis inhibitor.^5^

Bacterial escape from thrombi is further helped by T3SS, which, in contrast to Pla, not only prevents platelet aggregation but also promotes destabilization and disaggregation of already formed thrombi. Using a variety of mutants, the authors show that the T3SS injectosome itself, and not the effector proteins transmitted, can block initial platelet aggregation mediated by thrombin. Once the thrombus is formed, effector proteins YopH and YopE are essential to successfully destabilize the platelet clot. YopH and YopE affect cytoskeleton rearrangements in host cells,^6^ a process that is highly important during platelet aggregation.

YopH has similarities to eukaryotic protein tyrosine phosphatase, primarily targeting focal adhesion kinase and p130Cas, a docking protein that plays a central coordinating role for tyrosine kinase‐based signalling related to cell adhesion.^7^ Following its injection into the host cell, YopH rapidly dephosphorylates these cell adhesion‐promoting molecules that had become activated during the initial contact between the host cell and *Yersinia*. YopE acts as a GTPase‐activating protein toward the RhoA family of GTPases (RhoA, Rac, and Cdc42),^6^ thus accounting for the cytoskeletal collapse observed in *Yersinia*‐infected cells. Both proteins lead to disturbed adhesion and cytoskeleton rearrangements and therefore also contribute to the antiphagocytic activity of this pathogen.

The authors demonstrate that YopH and YopG change platelet morphology with formation of elongated, but barely branched lamellipodia, indicating that the T3SS system destabilizes thrombi by interfering with platelet cytoskeleton remodelling, which is in line with findings from other cellular systems.

In experiments with different *Y pestis* mutants, Palace et al discovered that platelet‐trapped bacteria were primarily killed by neutrophils and not by platelets, which also bear antimicrobial capacity. They further showed that neutrophils were less efficient in killing bacteria when they possess T3SS activity. The authors identified that the T3SS system enhances bacterial survival by enabling neutrophil extracellular trap (NET) formation, another host defence mechanism against tricky invaders. These insoluble NETs, consisting of expelled nuclear DNA and proteins, have one clear mission: to capture and kill bacteria.^8^ Platelets augment neutrophil NET formation and NETs in turn are highly pro‐thrombotic, highlighting the tight interplay between the hemostatic and the immune system.^1^, ^9^, ^10^ However, NETs also recruit and activate platelets by presenting histones to platelet toll‐like receptors (TLR).^11^, ^12^ Platelet TLRs mediate platelet activation in response to pathogen interactions, which in turn foster the formation of NETs as direct interaction of platelets with neutrophils leads to neutrophil activation and induces migration and NET formation. However, *Y pestis* lipopolysaccharide manages to evade recognition via TLR4,^13^ which represents another witty way to deceive the host.

Taken together, Palace et al confirm and extend the current knowledge on *Y pestis*‐derived virulence factors and add another important piece to the puzzle why this disease is so potent and deadly. They elegantly demonstrate that *Y pestis* impedes platelet activation and diminishes thrombi, thereby escaping entrapment and killing by neutrophils. This is the first demonstration that bacterial T3SS can interfere with platelet function and thrombus stability. It will be exciting to learn if also other pathogens, which also express T3SS, can diminish platelet responses as efficiently. Moreover, in vivo studies are warranted to fully unravel the consequences of these platelet‐bacteria interactions and translate the findings to the clinical situation. Understanding the underlying mechanisms of bacteria‐mediated platelet dysregulation could ultimately help to understand and control hemostatic imbalance in septic patients.

## CONFLICT OF INTEREST

The author declares no conflict of interest.
